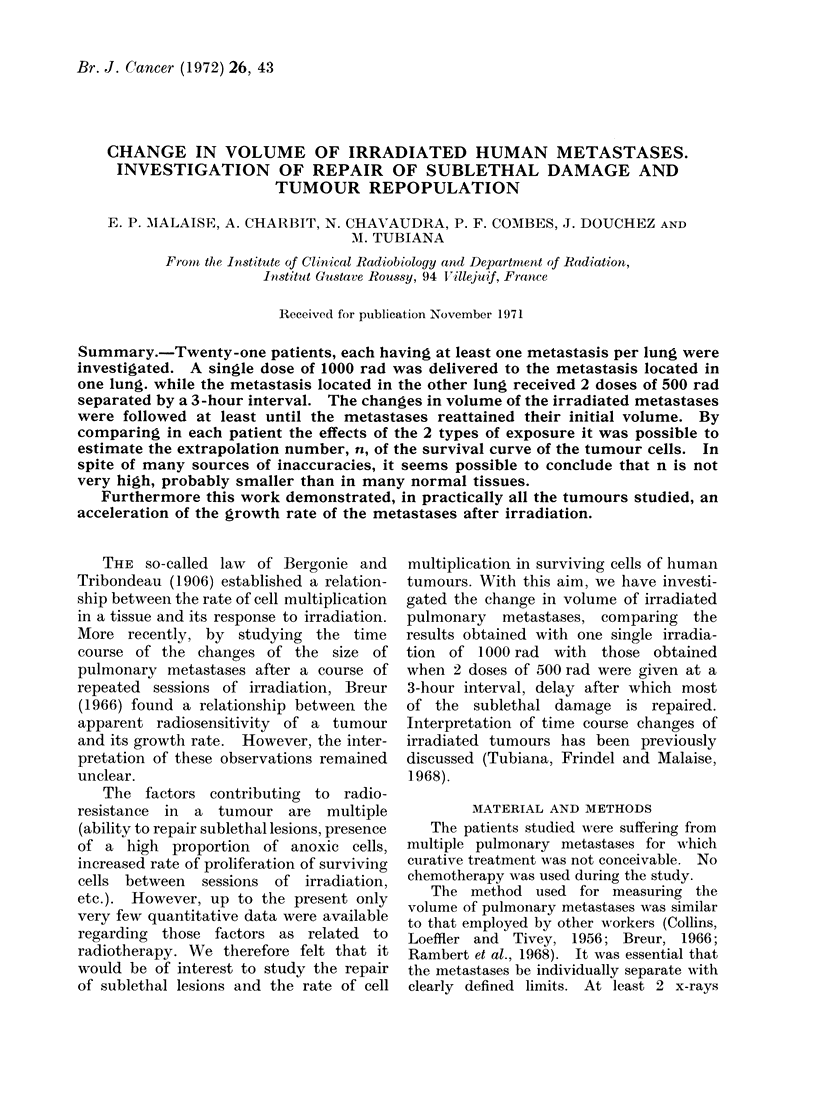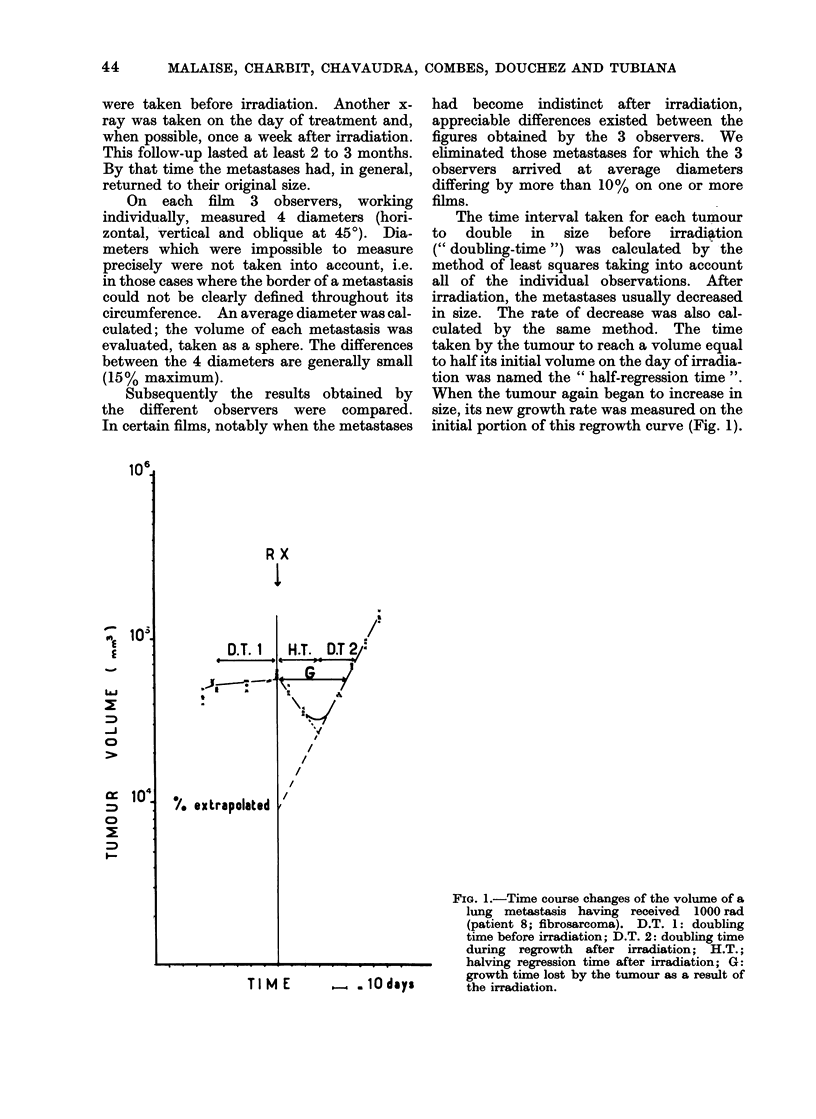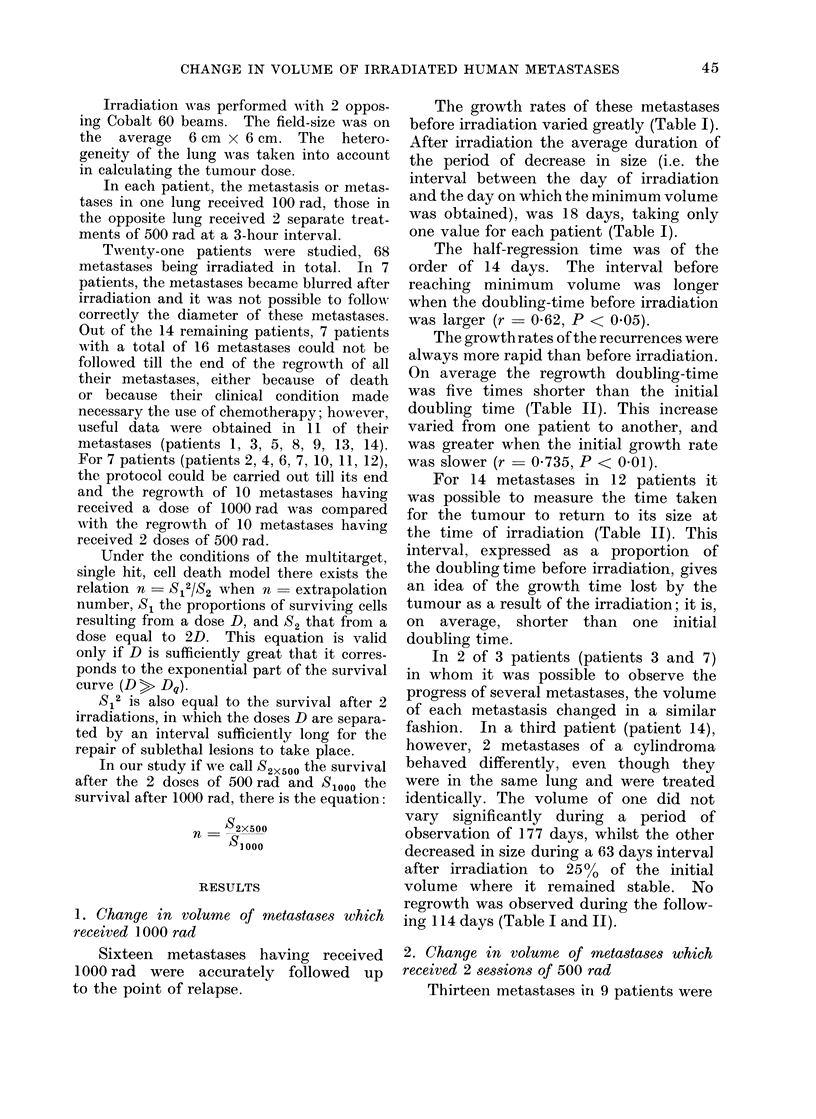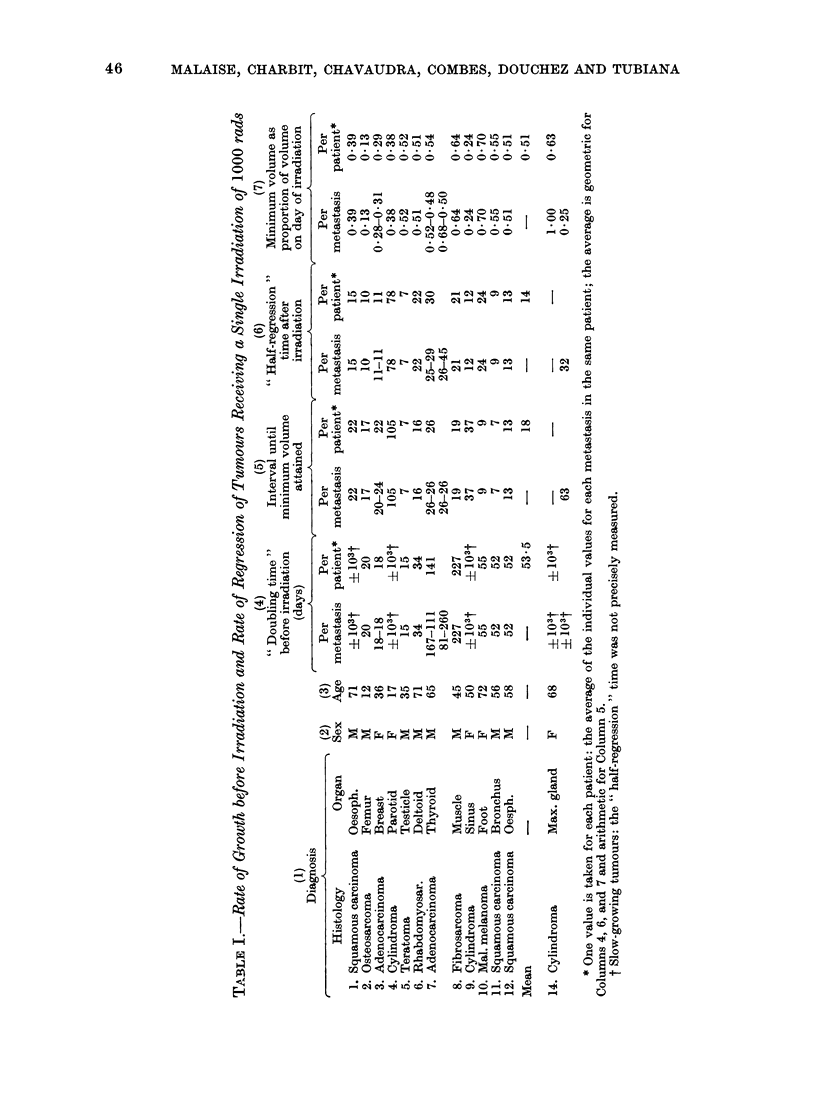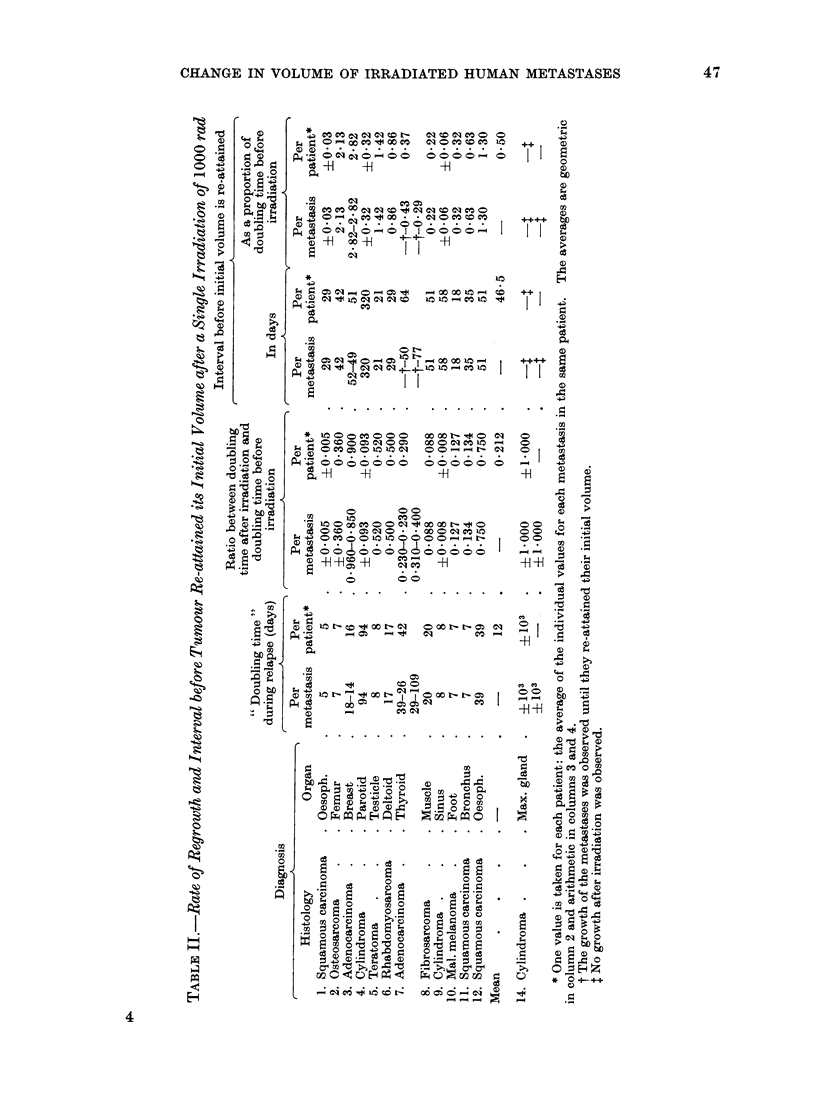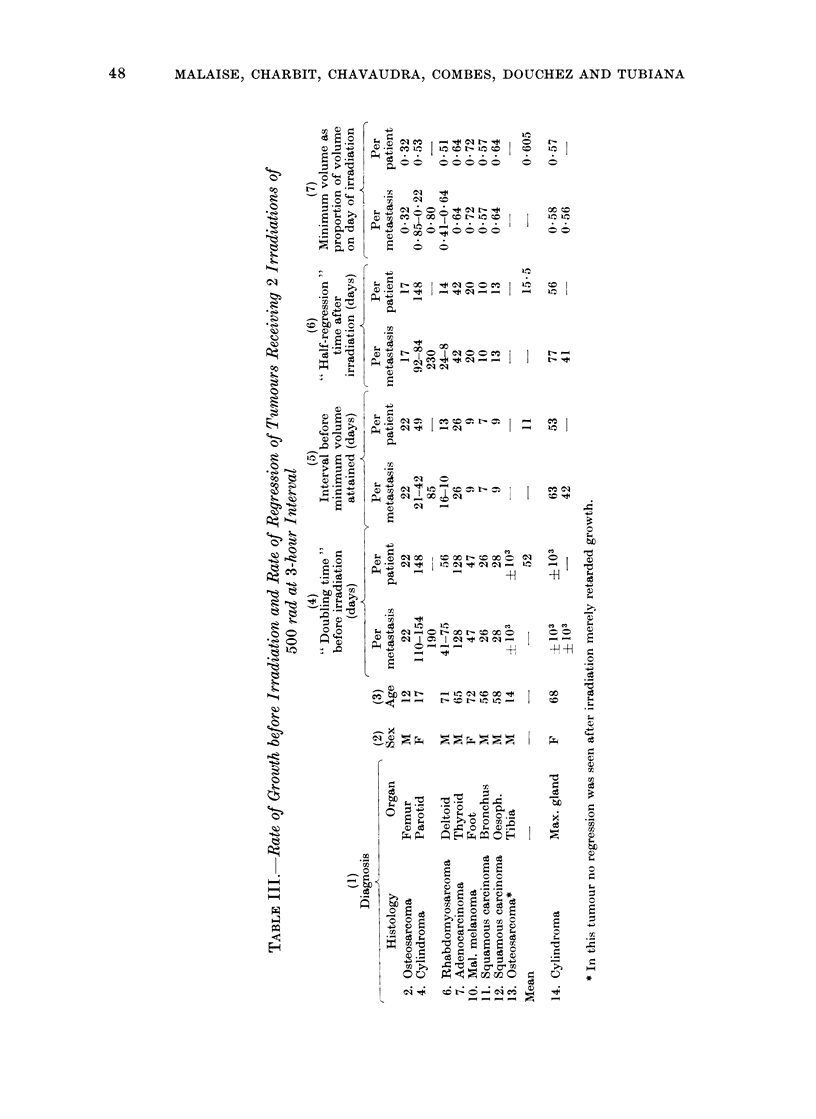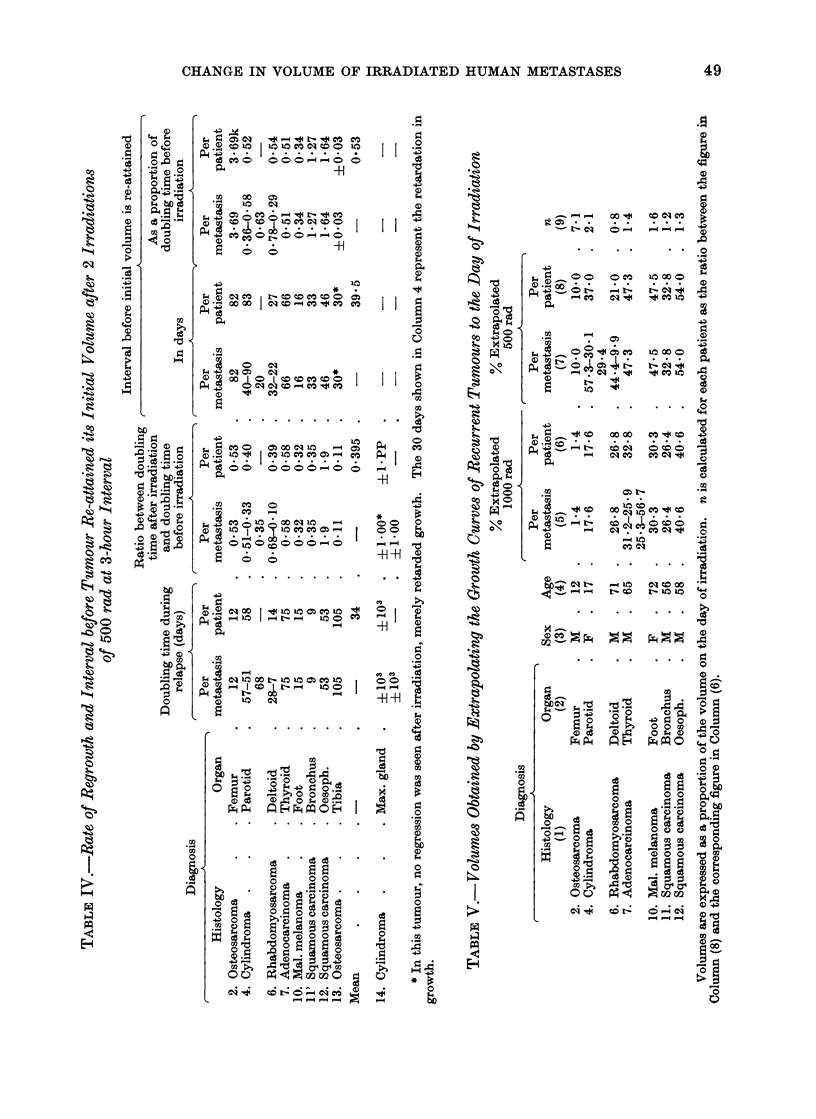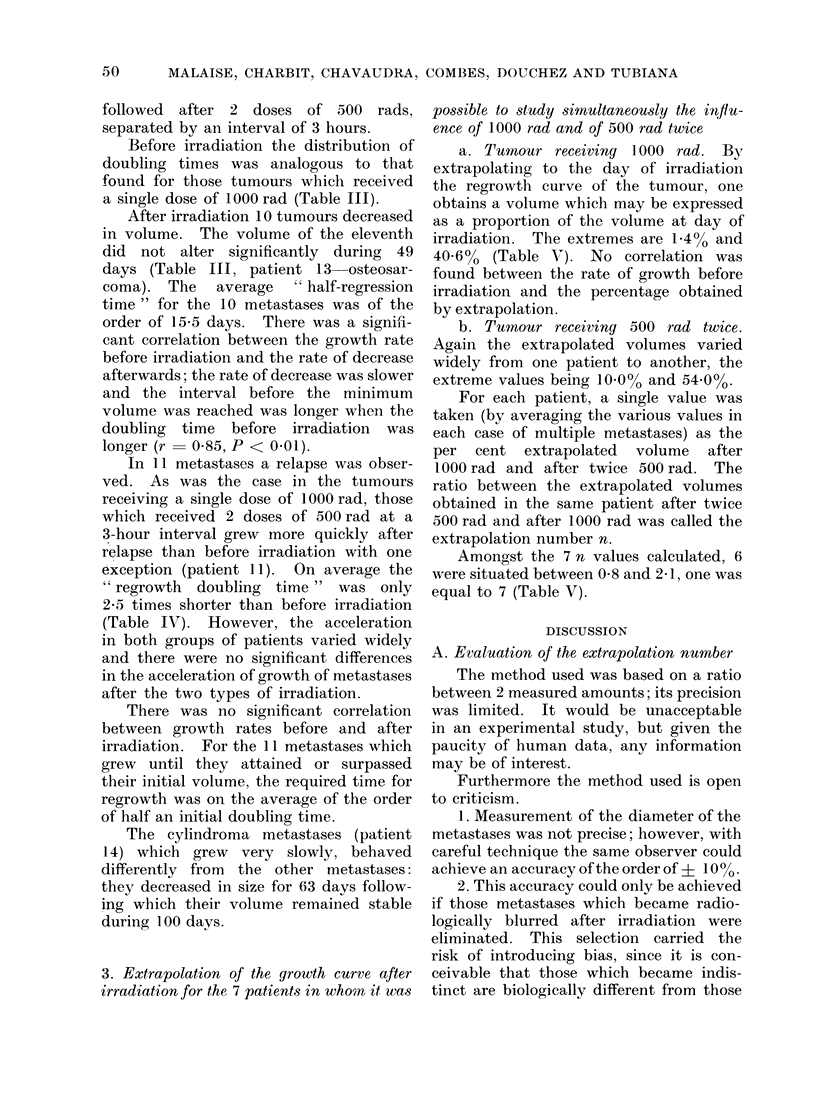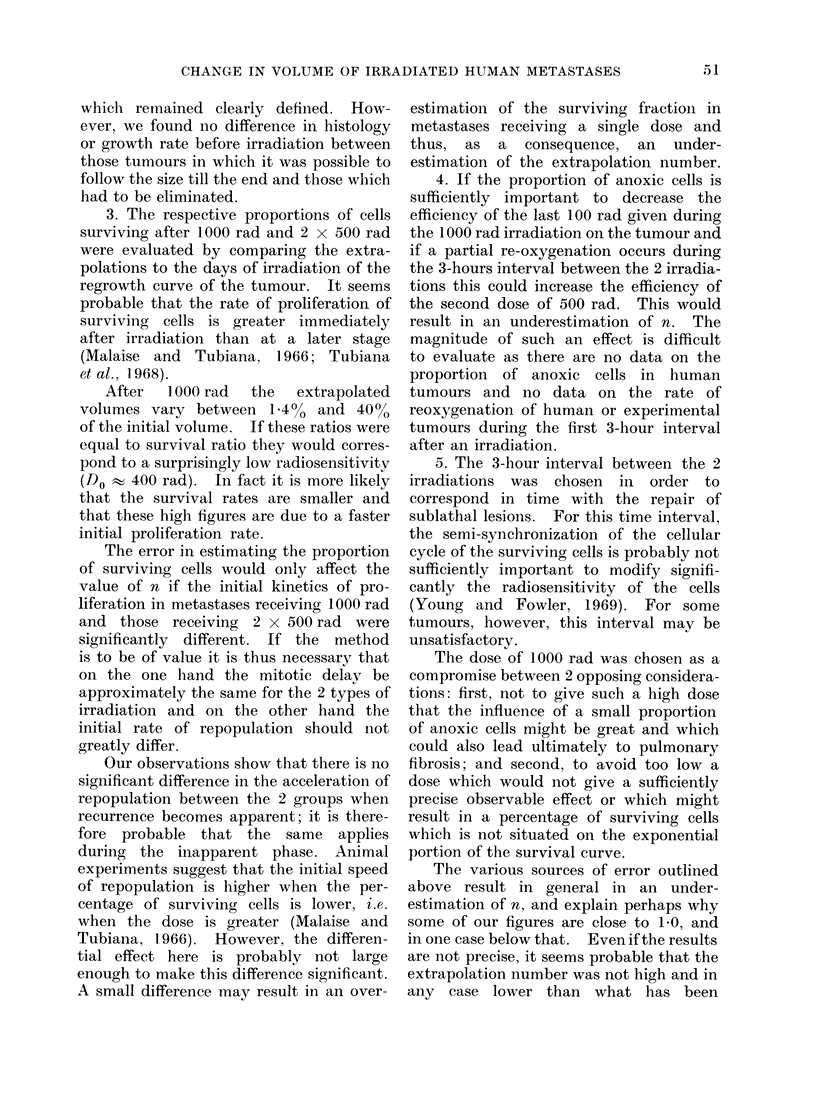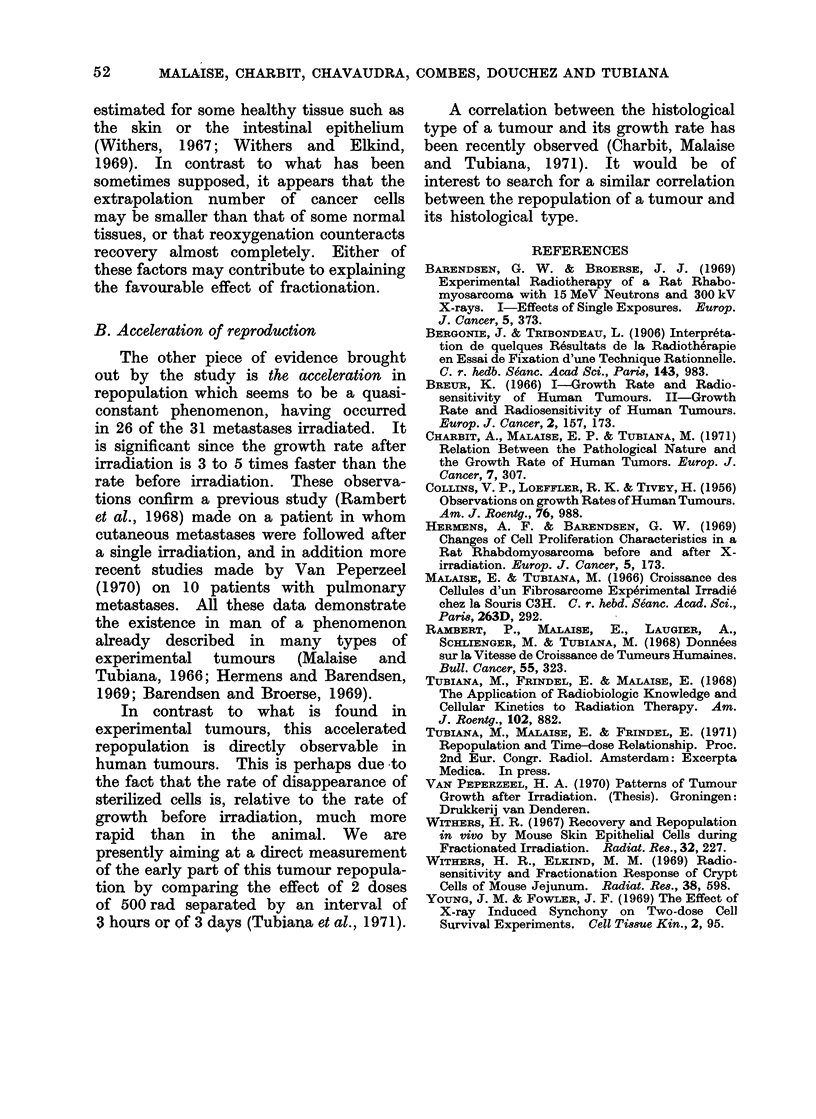# Change in Volume of Irradiated Human Metastases. Investigation of Repair of Sublethal Damage and Tumour Repopulation

**DOI:** 10.1038/bjc.1972.8

**Published:** 1972-02

**Authors:** E. P. Malaise, A. Charbit, N. Chavaudra, P. F. Combes, J. Douchez, M. Tubiana

## Abstract

Twenty-one patients, each having at least one metastasis per lung were investigated. A single dose of 1000 rad was delivered to the metastasis located in one lung. while the metastasis located in the other lung received 2 doses of 500 rad separated by a 3-hour interval. The changes in volume of the irradiated metastases were followed at least until the metastases reattained their initial volume. By comparing in each patient the effects of the 2 types of exposure it was possible to estimate the extrapolation number, *n,* of the survival curve of the tumour cells. In spite of many sources of inaccuracies, it seems possible to conclude that n is not very high, probably smaller than in many normal tissues.

Furthermore this work demonstrated, in practically all the tumours studied, an acceleration of the growth rate of the metastases after irradiation.


*Br. J. Cancer* (1972) **26**, 43

### CHANGE IN VOLUME OF IRRADIATED HUMAN METASTASES. INVESTIGATION OF REPAIR OF SUBLETHAL DAMAGE AND TUMOUR REPOPULATION

E. P. MALAISE, A. CHARBIT, N. CHAVAUDRA, P. F. COMBES, J. DOUCHEZ AND M. TUBIANA

*From the Institute of Clinical Radiobiology and Department of Radiation, Institut Gustave Roussy, 94 Villejuif, France*

Received for publication November 1971

**Summary.—Twenty-one patients, each having at least one metastasis per lung were investigated. A single dose of 1000 rad was delivered to the metastasis located in one lung. while the metastasis located in the other lung received 2 doses of 500 rad separated by a 3-hour interval. The changes in volume of the irradiated metastases were followed at least until the metastases reattained their initial volume. By comparing in each patient the effects of the 2 types of exposure it was possible to estimate the extrapolation number, *n*, of the survival curve of the tumour cells. In spite of many sources of inaccuracies, it seems possible to conclude that n is not very high, probably smaller than in many normal tissues.**

**Furthermore this work demonstrated, in practically all the tumours studied, an acceleration of the growth rate of the metastases after irradiation.**

THE so-called law of Bergonie and Tribondeau (1906) established a relationship between the rate of cell multiplication in a tissue and its response to irradiation. More recently, by studying the time course of the changes of the size of pulmonary metastases after a course of repeated sessions of irradiation, Breur (1966) found a relationship between the apparent radiosensitivity of a tumour and its growth rate. However, the interpretation of these observations remained unclear.

The factors contributing to radioresistance in a tumour are multiple (ability to repair sublethal lesions, presence of a high proportion of anoxic cells, increased rate of proliferation of surviving cells between sessions of irradiation, etc.). However, up to the present only very few quantitative data were available regarding those factors as related to radiotherapy. We therefore felt that it would be of interest to study the repair of sublethal lesions and the rate of cell multiplication in surviving cells of human tumours. With this aim, we have investigated the change in volume of irradiated pulmonary metastases, comparing the results obtained with one single irradiation of 1000 rad with those obtained when 2 doses of 500 rad were given at a 3-hour interval, delay after which most of the sublethal damage is repaired. Interpretation of time course changes of irradiated tumours has been previously discussed (Tubiana, Frindel and Malaise, 1968).

### MATERIAL AND METHODS

The patients studied were suffering from multiple pulmonary metastases for which curative treatment was not conceivable. No chemotherapy was used during the study.

The method used for measuring the volume of pulmonary metastases was similar to that employed by other workers (Collins, Loeffler and Tivey, 1956; Breur, 1966; Rambert *et al.*, 1968). It was essential that the metastases be individually separate with clearly defined limits. At least 2 x-rays were taken before irradiation. Another x-ray was taken on the day of treatment and, when possible, once a week after irradiation. This follow-up lasted at least 2 to 3 months. By that time the metastases had, in general, returned to their original size.

On each film 3 observers, working individually, measured 4 diameters (horizontal, vertical and oblique at 45°). Diameters which were impossible to measure precisely were not taken into account, i.e. in those cases where the border of a metastasis could not be clearly defined throughout its circumference. An average diameter was calculated; the volume of each metastasis was evaluated, taken as a sphere. The differences between the 4 diameters are generally small (15% maximum).

Subsequently the results obtained by the different observers were compared. In certain films, notably when the metastases had become indistinct after irradiation, appreciable differences existed between the figures obtained by the 3 observers. We eliminated those metastases for which the 3 observers arrived at average diameters differing by more than 10% on one or more films.

The time interval taken for each tumour to double in size before irradiation (" doubling-time ") was calculated by the method of least squares taking into account all of the individual observations. After irradiation, the metastases usually decreased in size. The rate of decrease was also calculated by the same method. The time taken by the tumour to reach a volume equal to half its initial volume on the day of irradiation was named the " half-regression time ". When the tumour again began to increase in size, its new growth rate was measured on the initial portion of this regrowth curve (Fig. 1).

Fig. 1.—Time course changes of the volume of a lung metastasis having received 1000 rad (patient 8; fibrosarcoma). D.T. 1: doubling time before irradiation; D.T. 2: doubling time during regrowth after irradiation; H.T.; halving regression time after irradiation; G: growth time lost by the tumour as a result of the irradiation.

Irradiation was performed with 2 opposing Cobalt 60 beams. The field-size was on the average 6 cm $\times$ 6 cm. The heterogeneity of the lung was taken into account in calculating the tumour dose.

In each patient, the metastasis or metastases in one lung received 100 rad, those in the opposite lung received 2 separate treatments of 500 rad at a 3-hour interval.

Twenty-one patients were studied, 68 metastases being irradiated in total. In 7 patients, the metastases became blurred after irradiation and it was not possible to follow correctly the diameter of these metastases. Out of the 14 remaining patients, 7 patients with a total of 16 metastases could not be followed till the end of the regrowth of all their metastases, either because of death or because their clinical condition made necessary the use of chemotherapy; however, useful data were obtained in 11 of their metastases (patients 1, 3, 5, 8, 9, 13, 14). For 7 patients (patients 2, 4, 6, 7, 10, 11, 12), the protocol could be carried out till its end and the regrowth of 10 metastases having received a dose of 1000 rad was compared with the regrowth of 10 metastases having received 2 doses of 500 rad.

Under the conditions of the multitarget, single hit, cell death model there exists the relation $n = S_1^2/S_2$ when $n =$ extrapolation number, $S_1$ the proportions of surviving cells resulting from a dose $D$, and $S_2$ that from a dose equal to $2D$. This equation is valid only if $D$ is sufficiently great that it corresponds to the exponential part of the survival curve ($D \gg D_q$).

$S_1^2$ is also equal to the survival after 2 irradiations, in which the doses $D$ are separated by an interval sufficiently long for the repair of sublethal lesions to take place.

In our study if we call $S_{2\times500}$ the survival after the 2 doses of 500 rad and $S_{1000}$ the survival after 1000 rad, there is the equation:

$$n = \frac{S_{2\times500}}{S_{1000}}$$

### RESULTS

### 1. *Change in volume of metastases which received* 1000 *rad*

Sixteen metastases having received 1000 rad were accurately followed up to the point of relapse.

The growth rates of these metastases before irradiation varied greatly (Table I). After irradiation the average duration of the period of decrease in size (i.e. the interval between the day of irradiation and the day on which the minimum volume was obtained), was 18 days, taking only one value for each patient (Table I).

The half-regression time was of the order of 14 days. The interval before reaching minimum volume was longer when the doubling-time before irradiation was larger ($r = 0{\cdot}62$, $P < 0{\cdot}05$).

The growth rates of the recurrences were always more rapid than before irradiation. On average the regrowth doubling-time was five times shorter than the initial doubling time (Table II). This increase varied from one patient to another, and was greater when the initial growth rate was slower ($r = 0{\cdot}735$, $P < 0{\cdot}01$).

For 14 metastases in 12 patients it was possible to measure the time taken for the tumour to return to its size at the time of irradiation (Table II). This interval, expressed as a proportion of the doubling time before irradiation, gives an idea of the growth time lost by the tumour as a result of the irradiation; it is, on average, shorter than one initial doubling time.

In 2 of 3 patients (patients 3 and 7) in whom it was possible to observe the progress of several metastases, the volume of each metastasis changed in a similar fashion. In a third patient (patient 14), however, 2 metastases of a cylindroma behaved differently, even though they were in the same lung and were treated identically. The volume of one did not vary significantly during a period of observation of 177 days, whilst the other decreased in size during a 63 days interval after irradiation to 25% of the initial volume where it remained stable. No regrowth was observed during the following 114 days (Table I and II).

### 2. *Change in volume of metastases which received* 2 *sessions of* 500 *rad*

Thirteen metastases in 9 patients were followed after 2 doses of 500 rads, separated by an interval of 3 hours.

Before irradiation the distribution of doubling times was analogous to that found for those tumours which received a single dose of 1000 rad (Table III).

After irradiation 10 tumours decreased in volume. The volume of the eleventh did not alter significantly during 49 days (Table III, patient 13—osteosarcoma). The average " half-regression time " for the 10 metastases was of the order of 15·5 days. There was a significant correlation between the growth rate before irradiation and the rate of decrease afterwards; the rate of decrease was slower and the interval before the minimum volume was reached was longer when the doubling time before irradiation was longer ($r = 0{\cdot}85$, $P < 0{\cdot}01$).

In 11 metastases a relapse was observed. As was the case in the tumours receiving a single dose of 1000 rad, those which received 2 doses of 500 rad at a 3-hour interval grew more quickly after relapse than before irradiation with one exception (patient 11). On average the " regrowth doubling time " was only 2·5 times shorter than before irradiation (Table IV). However, the acceleration in both groups of patients varied widely and there were no significant differences in the acceleration of growth of metastases after the two types of irradiation.

There was no significant correlation between growth rates before and after irradiation. For the 11 metastases which grew until they attained or surpassed their initial volume, the required time for regrowth was on the average of the order of half an initial doubling time.

The cylindroma metastases (patient 14) which grew very slowly, behaved differently from the other metastases: they decreased in size for 63 days following which their volume remained stable during 100 days.

3. *Extrapolation of the growth curve after irradiation for the* 7 *patients in whom it was possible to study simultaneously the influence of* 1000 *rad and of* 500 *rad twice*

a. *Tumour receiving* 1000 *rad.* By extrapolating to the day of irradiation the regrowth curve of the tumour, one obtains a volume which may be expressed as a proportion of the volume at day of irradiation. The extremes are 1·4% and 40·6% (Table V). No correlation was found between the rate of growth before irradiation and the percentage obtained by extrapolation.

b. *Tumour receiving* 500 *rad twice.* Again the extrapolated volumes varied widely from one patient to another, the extreme values being 10·0% and 54·0%.

For each patient, a single value was taken (by averaging the various values in each case of multiple metastases) as the per cent extrapolated volume after 1000 rad and after twice 500 rad. The ratio between the extrapolated volumes obtained in the same patient after twice 500 rad and after 1000 rad was called the extrapolation number $n$.

Amongst the 7 $n$ values calculated, 6 were situated between 0·8 and 2·1, one was equal to 7 (Table V).

### DISCUSSION

### A. *Evaluation of the extrapolation number*

The method used was based on a ratio between 2 measured amounts; its precision was limited. It would be unacceptable in an experimental study, but given the paucity of human data, any information may be of interest.

Furthermore the method used is open to criticism.

1. Measurement of the diameter of the metastases was not precise; however, with careful technique the same observer could achieve an accuracy of the order of $\pm$ 10%.

2. This accuracy could only be achieved if those metastases which became radiologically blurred after irradiation were eliminated. This selection carried the risk of introducing bias, since it is conceivable that those which became indistinct are biologically different from those which remained clearly defined. However, we found no difference in histology or growth rate before irradiation between those tumours in which it was possible to follow the size till the end and those which had to be eliminated.

3. The respective proportions of cells surviving after 1000 rad and 2 × 500 rad were evaluated by comparing the extrapolations to the days of irradiation of the regrowth curve of the tumour. It seems probable that the rate of proliferation of surviving cells is greater immediately after irradiation than at a later stage (Malaise and Tubiana, 1966; Tubiana *et al.*, 1968).

After 1000 rad the extrapolated volumes vary between 1·4% and 40% of the initial volume. If these ratios were equal to survival ratio they would correspond to a surprisingly low radiosensitivity ($D_0 \approx 400$ rad). In fact it is more likely that the survival rates are smaller and that these high figures are due to a faster initial proliferation rate.

The error in estimating the proportion of surviving cells would only affect the value of $n$ if the initial kinetics of proliferation in metastases receiving 1000 rad and those receiving 2 × 500 rad were significantly different. If the method is to be of value it is thus necessary that on the one hand the mitotic delay be approximately the same for the 2 types of irradiation and on the other hand the initial rate of repopulation should not greatly differ.

Our observations show that there is no significant difference in the acceleration of repopulation between the 2 groups when recurrence becomes apparent; it is therefore probable that the same applies during the inapparent phase. Animal experiments suggest that the initial speed of repopulation is higher when the percentage of surviving cells is lower, *i.e.* when the dose is greater (Malaise and Tubiana, 1966). However, the differential effect here is probably not large enough to make this difference significant. A small difference may result in an overestimation of the surviving fraction in metastases receiving a single dose and thus, as a consequence, an underestimation of the extrapolation number.

4. If the proportion of anoxic cells is sufficiently important to decrease the efficiency of the last 100 rad given during the 1000 rad irradiation on the tumour and if a partial re-oxygenation occurs during the 3-hours interval between the 2 irradiations this could increase the efficiency of the second dose of 500 rad. This would result in an underestimation of $n$. The magnitude of such an effect is difficult to evaluate as there are no data on the proportion of anoxic cells in human tumours and no data on the rate of reoxygenation of human or experimental tumours during the first 3-hour interval after an irradiation.

5. The 3-hour interval between the 2 irradiations was chosen in order to correspond in time with the repair of sublathal lesions. For this time interval, the semi-synchronization of the cellular cycle of the surviving cells is probably not sufficiently important to modify significantly the radiosensitivity of the cells (Young and Fowler, 1969). For some tumours, however, this interval may be unsatisfactory.

The dose of 1000 rad was chosen as a compromise between 2 opposing considerations: first, not to give such a high dose that the influence of a small proportion of anoxic cells might be great and which could also lead ultimately to pulmonary fibrosis; and second, to avoid too low a dose which would not give a sufficiently precise observable effect or which might result in a percentage of surviving cells which is not situated on the exponential portion of the survival curve.

The various sources of error outlined above result in general in an underestimation of $n$, and explain perhaps why some of our figures are close to 1·0, and in one case below that. Even if the results are not precise, it seems probable that the extrapolation number was not high and in any case lower than what has been estimated for some healthy tissue such as the skin or the intestinal epithelium (Withers, 1967; Withers and Elkind, 1969). In contrast to what has been sometimes supposed, it appears that the extrapolation number of cancer cells may be smaller than that of some normal tissues, or that reoxygenation counteracts recovery almost completely. Either of these factors may contribute to explaining the favourable effect of fractionation.

### *B. Acceleration of reproduction*

The other piece of evidence brought out by the study is *the acceleration* in repopulation which seems to be a quasi-constant phenomenon, having occurred in 26 of the 31 metastases irradiated. It is significant since the growth rate after irradiation is 3 to 5 times faster than the rate before irradiation. These observations confirm a previous study (Rambert *et al.*, 1968) made on a patient in whom cutaneous metastases were followed after a single irradiation, and in addition more recent studies made by Van Peperzeel (1970) on 10 patients with pulmonary metastases. All these data demonstrate the existence in man of a phenomenon already described in many types of experimental tumours (Malaise and Tubiana, 1966; Hermens and Barendsen, 1969; Barendsen and Broerse, 1969).

In contrast to what is found in experimental tumours, this accelerated repopulation is directly observable in human tumours. This is perhaps due to the fact that the rate of disappearance of sterilized cells is, relative to the rate of growth before irradiation, much more rapid than in the animal. We are presently aiming at a direct measurement of the early part of this tumour repopulation by comparing the effect of 2 doses of 500 rad separated by an interval of 3 hours or of 3 days (Tubiana *et al.*, 1971).

A correlation between the histological type of a tumour and its growth rate has been recently observed (Charbit, Malaise and Tubiana, 1971). It would be of interest to search for a similar correlation between the repopulation of a tumour and its histological type.

### REFERENCES

BARENDSEN, G. W. & BROERSE, J. J. (1969) Experimental Radiotherapy of a Rat Rhabomyosarcoma with 15 MeV Neutrons and 300 kV X-rays. I—Effects of Single Exposures. *Europ. J. Cancer*, **5**, 373.

BERGONIE, J. & TRIBONDEAU, L. (1906) Interprétation de quelques Résultats de la Radiothérapie en Essai de Fixation d'une Technique Rationnelle. *C. r. hedb. Séanc. Acad Sci., Paris*, **143**, 983.

BREUR, K. (1966) I—Growth Rate and Radiosensitivity of Human Tumours. II—Growth Rate and Radiosensitivity of Human Tumours. *Europ. J. Cancer*, **2**, 157, 173.

CHARBIT, A., MALAISE, E. P. & TUBIANA, M. (1971) Relation Between the Pathological Nature and the Growth Rate of Human Tumors. *Europ. J. Cancer*, **7**, 307.

COLLINS, V. P., LOEFFLER, R. K. & TIVEY, H. (1956) Observations on growth Rates of Human Tumours. *Am. J. Roentg.*, **76**, 988.

HERMENS, A. F. & BARENDSEN, G. W. (1969) Changes of Cell Proliferation Characteristics in a Rat Rhabdomyosarcoma before and after X-irradiation. *Europ. J. Cancer*, **5**, 173.

MALAISE, E. & TUBIANA, M. (1966) Croissance des Cellules d'un Fibrosarcome Expérimental Irradié chez la Souris C3H. *C. r. hebd. Séanc. Acad. Sci., Paris*, **263D**, 292.

RAMBERT, P., MALAISE, E., LAUGIER, A., SCHLIENGER, M. & TUBIANA, M. (1968) Données sur la Vitesse de Croissance de Tumeurs Humaines. *Bull. Cancer*, **55**, 323.

TUBIANA, M., FRINDEL, E. & MALAISE, E. (1968) The Application of Radiobiologic Knowledge and Cellular Kinetics to Radiation Therapy. *Am. J. Roentg.*, **102**, 882.

TUBIANA, M., MALAISE, E. & FRINDEL, E. (1971) Repopulation and Time–dose Relationship. Proc. 2nd Eur. Congr. Radiol. Amsterdam: Excerpta Medica. In press.

VAN PEPERZEEL, H. A. (1970) Patterns of Tumour Growth after Irradiation. (Thesis). Groningen: Drukkerij van Denderen.

WITHERS, H. R. (1967) Recovery and Repopulation *in vivo* by Mouse Skin Epithelial Cells during Fractionated Irradiation. *Radiat. Res.*, **32**, 227.

WITHERS, H. R., ELKIND, M. M. (1969) Radiosensitivity and Fractionation Response of Crypt Cells of Mouse Jejunum. *Radiat. Res.*, **38**, 598.

YOUNG, J. M. & FOWLER, J. F. (1969) The Effect of X-ray Induced Synchony on Two-dose Cell Survival Experiments. *Cell Tissue Kin.*, **2**, 95.